# Influence of Defects on Bending Properties of 2D-T700/E44 Composites Prepared by Improved Compression Molding Process

**DOI:** 10.3390/ma11112132

**Published:** 2018-10-30

**Authors:** Yuqin Ma, Shuangshuang Li, Jie Wang, Luyan Ju, Xinmei Liu

**Affiliations:** 1 School of Mechano-Electronic Engineering, Xidian University, Xi’an 710071, China; 15238166225@163.com (S.L.); imustwj@163.com (J.W.); liuxinmei3254@163.com (X.L.); 2 Mechanical Engineering College, Xi’an Shiyou University, Xi’an 710065, China

**Keywords:** improved compression molding process, T700/E44 composite materials, defects, microstructure, bending property

## Abstract

2D-T700/E44 composite materials were prepared by improved compression molding process (ICM) then microstructure and properties of the composites were analyzed and summarized by scanning electron microscope (SEM) and electronic universal testing machine. It is found that defects will occur when the process parameters are not controlled properly and the main defects of composite materials include inadequate resin impregnation, weak interlaminar binding force, fiber displacement warping, hole and brittle fracture. Moreover, there are significant differences in the infiltration microstructure, bending properties, and fracture morphology of the composite materials with different defects. When the defects of weak interlaminar binding force and brittle fracture occur, bending properties of composite materials are relatively low, and they are 220 MPa and 245 MPa, respectively, which reach 34.9% and 38.9% of the bending strength of composite material whose defects are effectively controlled. When the process parameters are reasonable and the defects of the composite materials are effectively eliminated, the bending strength can reach 630 MPa. This will lay a foundation for the preparation of 2D-T700/E44 composite materials with ideal microstructures and properties by ICM.

## 1. Introduction

Fiber-reinforced composites have a series of excellent properties, such as low density, high specific strength, and specific stiffness, low wear resistance, low thermal expansion coefficient, good fatigue resistance, good dimensional stability, etc. They have wide application prospects in aerospace, weapon equipment, communications electronics and transportation [1,2,3,4]. Carbon fiber-reinforced resin matrix composites (CFRP) have received research and attention from more and more people. Moreover, two-dimensional (2D) woven carbon fiber-reinforced resin matrix composites can guarantee the consistency properties of composites in the directions of 2D fibers and overcome the limitation of unidirectional (1D) carbon fiber-reinforced resin matrix composites, which have superior properties only in the direction of single fibers [5,6]. Therefore, the research and application of carbon fiber-reinforced resin matrix composites with 2D woven structure are more and more extensive.

At present, the preparation methods of carbon fiber-reinforced resin matrix composites mainly include hand paste molding, winding molding, extrusion molding, resin transfer molding and molding process [7,8,9,10]. However, the preparation efficiency of hand paste molding is low and the forming pressure is low, and the preparation defects such as holes and stratification are easy to appear inside the composite materials. The equipment of winding molding, extrusion molding and resin transfer molding is relatively complex and has high cost. The traditional molding process is to put the carbon fiber and impregnates fiber material into the metal mold. After heating, pressing and solidifying, the resin material can be plasticized and flow, and then fills the mold cavity. The compacted composite material will be obtained finally. The composite material prepared by this process has good microstructure and good performance, but the viscous resin has good fluidity when it is pressed at a high temperature, and it is easy to adhere to the mold. For the laminated carbon fiber-reinforced resin matrix composites, the bonding force between the carbon fiber layers is very small when resin is at a high temperature, so defects of fiber segregation and displacement will occur easily. This will affect the microstructure and mechanical properties of laminated composite materials seriously. Meanwhile, the viscous resin with good fluidity will also make it difficult to take out the composites. All of these bring large difficulties to the preparation of the ideal composite with microstructures and property.

In this paper, an improved experimental system of improved compression molding process (ICM) is proposed on the basis of traditional molding method. Firstly, the precut and impregnated fiber material are put into the high temperature curing box to preliminarily realize the flow and impregnation of the resin. Then, after the initial curing of the resin, it is transferred to the hot pressing mold to complete the final curing of the composite material. This will prevent the resin from sticking to the mold so that it is easy to take out the parts, and fiber segregation and displacement defects do not occur easily, which are beneficial to the preparation of 2D-T700/E44 composite with high properties. However, when this process is used to prepare composite materials, it involves parameters such as solution ratio, curing temperature, pressure and time, etc. When the process parameters are not controlled properly, it is easy to produce defects, which will affect the successful preparation of composite materials with ideal microstructure and performance. Some scholars have studied the fabrication defects of composites [11,12,13], but influence of different defects on bending properties of 2D-T700/E44 composites prepared by ICM has not been reported. It is very necessary to carry out defect research of laminated 2D-T700/E44 composites.

In this paper, 2D-T700/E44 composite materials were prepared by ICM. Then combined with microstructure observation analysis and bending strength test, the defects such as insufficient impregnation of solidified mixed solution, weak interlaminar binding force, fiber displacement warping, pore hole and brittle fracture of composite materials during the preparation of composite materials were thoroughly analyzed. Finally, the main causes of defects and the control measures to avoid defects were obtained, which would lay a foundation for the successful preparation of ideal composite materials.

## 2. Experimental Materials and Methods

### 2.1. Experimental Materials

The reinforcement used in the study is 12K unidirectional T700 carbon fiber cloth produced by Toray in Japan, and the matrix used is E44 epoxy resin produced by Xi’an resin factory in China. The curing agent used is 593 curing agent of Sanmu Group Co. Ltd. (Yi Xing, China)

### 2.2. Experimental Methods

**Figure 1:**
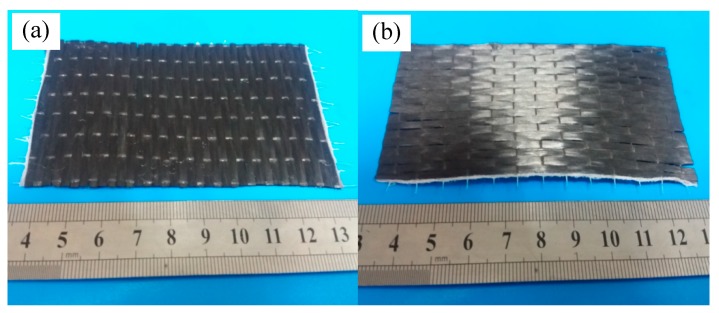
Cutting T700 carbon fiber cloth sheets with the two fiber directions. (**a**) Length direction is perpendicular to the fiber axis; (**b**)The length direction is parallel to the fiber axis.

In the experiment, T700 carbon fiber non-woven fabric was cut into rectangular thin sections, and its length and width were 100 mm and 50 mm, respectively. The cutting carbon fiber cloth with the two fiber directions was shown in Figure 1.

**Figure 2:**
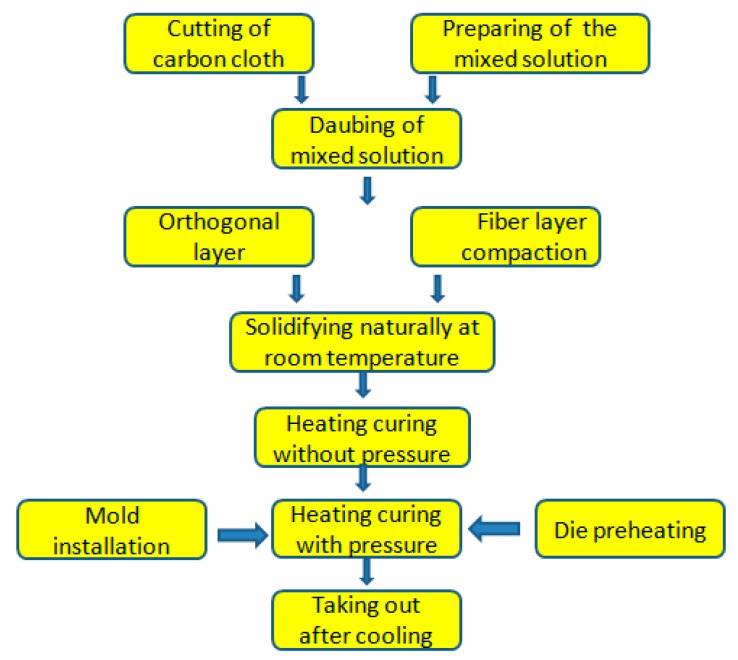
Process of preparing 2D-T700/E44 composite materials by ICM.

2D-T700/E44 composite materials were prepared by ICM. The steps of using this process to prepare 2D-T700/E44 composite materials are as follows. (1) E-44 epoxy resin and 593 curing agent were mixed into a curing mixed solution according to the mass ratio of 5:1. (2) The curing mixed solution was spread on both sides of the carbon fiber cloth sheets, and then sheets were laminated and compacted. The rotation angle between adjacent fiber laminations is 90°, and lamination thickness of the composites prepared is 2~2.5 mm. (3) Carbon fiber laminations were placed at room temperature under the condition of 6 h to achieve natural curing process, and then they were transferred to the constant temperature drying box to achieve pressure-free heating curing process, whose curing temperature and time were 115 °C and 110 min, respectively. (4) The carbon fiber lamination was taken out from the constant temperature drying box and put into a preheated hot pressing mold to achieve pressure heating curing process. The curing temperature and pressure were 50 °C and 10 MPa respectively, and pressure holding time was 25 min. (5) The heating and pressure were stopped. After the mold was restored to room temperature, 2D-T700/E44 composite material was obtained by taking out from the mold. The process of preparing 2D-T700/E44 composite materials by ICM was shown in Figure 2, and the main process parameters adopted were shown in Table 1.

### 2.3. Testing and Characterization Methods

**Figure 3:**
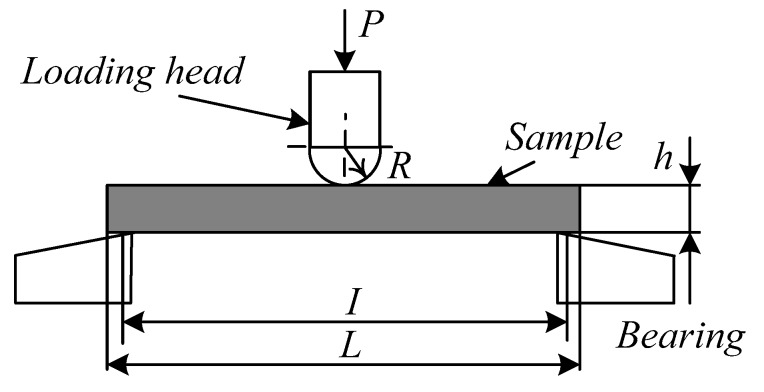
Three-point bending test method diagram. *P*—Load, *I*—Sample span, *L*—Length of the sample, *h*—Sample thickness, *R*—Radius of the loading head.

The impregnation microstructure and bending fracture morphology of 2D-T700/E44 composite material were observed by JEOL JSM-6390A scanning electron microscope (SEM). The bending strength of 2D-T700/E44 composite was tested by DNS100 electronic universal testing machine of Changchun institute of mechanical science and the test method followed Test Method for Flexural Properties of Fiber-reinforced Plastics (GB/T1449-2005). The bending sample size is 50 mm × 15 mm × 2 mm, and the loading upper head radius R is 5 mm. To obtain reasonable bending strength value of each composite, three bending strength specimens are tested, and representative bending strength of the composite is chosen in this study. The test loading speed is 10 mm/min, and the span is 40 mm. The experimental schematic diagram is shown in Figure 3.

## 3. Experimental Results and Analysis

The improved compression molding process (ICM) for the preparation of 2D-T700/E44 composites involves the processes of preparation of the curing mixed solution, coating on the carbon fiber cloth of the curing mixed solution, natural curing, pressure-free heating curing, and pressure heating curing. It also involves process parameters such as curing mixed ratio, natural curing time, pressure-free heating curing temperature and time, pressure heating curing pressure, and pressure heating curing temperature and time. All these process parameters can affect the preparation effect of 2D-T700/E44 composite materials, and defects such as insufficient resin impregnation, weak interlaminar binding force, fiber displacement warping, hole and brittle fracture of composite materials will occur, when they are not properly controlled. Therefore, it is necessary to conduct in-depth study on the causes of these defects and the control methods of reducing the defects to prepare 2D-T700/E44 composite materials with ideal microstructures and properties.

### 3.1. Insufficient Resin Impregnation

**Figure 4:**
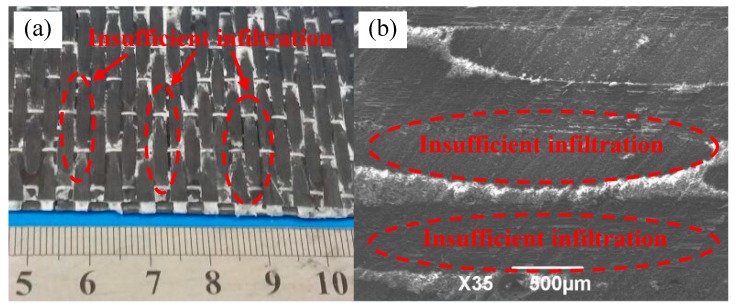
Imperfection of impregnation of 2D-T700/E44 composite prepared by ICM (**a**) Macrograph of imperfection impregnation; (**b**) Micrograph of imperfection impregnation.

When the curing temperature and pressure are not appropriate, the insufficient resin impregnation occurs easily [2]. Figure 4a shows the obvious imperfection of impregnation due to the insufficiency of curing temperature, pressure and other parameters. Its pressure-free heating curing temperature is 90 °C, and pressure heating curing temperature and pressure are 40 °C and 6 MPa respectively. There are significant gaps between carbon fiber bundles and the bundles (Figure 4a). Scanning electron microscope (SEM) was used to further observe and analyze the microstructure of the composite material (Figure 4b). Dark color is carbon fiber, and white color is resin. The presence of resin is rarely seen in the fiber bundle. Due to the bad adequacy and uniformity of resin impregnation, carbon fiber cannot play an effective role in strengthening, and the resin matrix cannot fully play the role of transferring load, which will affect the properties of composite materials. In Figure 4, the bending performance of the composite material with incomplete impregnation was tested, and the bending strength was 315 MPa, which was significantly improved than the resin matrix, but was not ideal. The impregnation effect of composite materials should be improved by increasing the pressure-free heating curing temperature, pressure heating curing temperature and pressure.

### 3.2. Weak Interlaminar Binding Force

**Figure 5:**
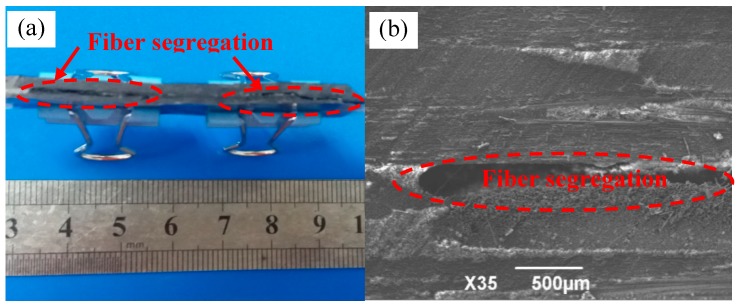
The defect of weak interlaminar binding force of 2D-T700/E44 composite prepared by ICM. (**a**) Macrograph defect; (**b**) Micrograph defect.

When the process parameters such as the ratio of the curing mixture solution or the curing temperature and pressure are inappropriate, the bonding force between the layers will be weak, so the properties of the materials will be reduced due to the lamination under loads. Figure 5 is the 2D-T700/E44 composite materials with weak interlaminar binding force which is prepared by ICM. The mass ratio of E44 epoxy resin and 593 curing agent was 6:1, and pressure-free heating curing temperature was 80 °C. Pressure heating curing temperature and pressure are 35 °C and 5 MPa respectively. From the Figure 5a, there isobvious fiber laminationin the composite. When the composite material conducts three-point bending test on the universal testing machine, the position with the weakest binding force between layers will be damaged first. Cracks will appear between layers, and gradually begin to expand. A long and narrow crack will appear at last.

As shown in the microscopic picture of fiber stratification (Figure 5b), it can be seen that the continuity between fiber and resin matrix is poor, due to the existence of fiber segregation. Fiber stratification will destroy the continuity of matrix in the composite, so matrix cannot effectively transfer the load, which affects the mechanical properties of the composite material. Through the bending property test, its bending strength was only 220 MPa, and its mechanical performance was not satisfactory. The defects of weak binding force between 2D-T700/E44 composite layers should be avoided by improving the mass ratio of resin and curing agent, increasing the curing temperature and increasing the curing pressure.

### 3.3. Fiber Displacement Warping

**Figure 6:**
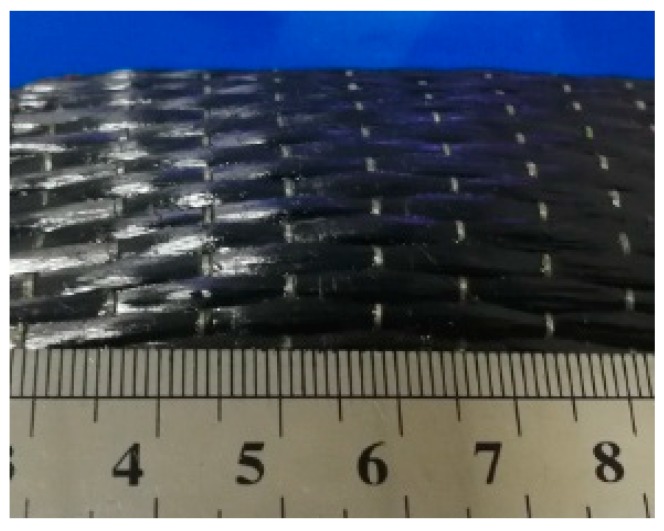
The defect of fiber displacement warping of 2D-T700/E44 composite prepared by ICM.

When 2D-T700/E44 composite materials were prepared by ICM, if the curing temperature and time were not reasonable, fiber displacement or warping would occur, and the carbon fiber in the same layer was not distributed in a plane (Figure 6). Its pressure-free heating curing time and temperature are 140 min and 140 °C respectively. In addition, during the curing process of composite materials, the bending carbon fiber has been subjected to stress, and local damage will be caused, which will damage and decrease the carrying capacity of the carbon fiber cloth. When the composite material is subjected to bending load again, it is easy to break and damage, and the bending properties of the composite materials will decrease [14]. By testing the bending properties of composites with fiber warping defects, the bending strength of the composites was 260 MPa, and the mechanical properties were not satisfactory. This kind of defect should be avoided by reducing the temperature and time of pressure-free heating curing time and temperature.

### 3.4. Hole

When 2D-T700/E44 composite materials were prepared by ICM, hole defects would appear in the composites if the pressure, temperature and time of pressure heating curing were not feasible. The hole is mainly formed by gas accumulation which is not eliminated in time, which leads to irregular shape and size under load. The discharge of gas is affected by the fluidity of the curing mixed solution, and fluidity is influenced by its temperature. If the temperature of curing mixed solution is too low, its fluidity is poor, and the gas discharge rate is slow, so bubbles occur easily. If the temperature is too high, the mixture has been solidified before gas discharges, and this will result in hole. 

**Figure 7:**
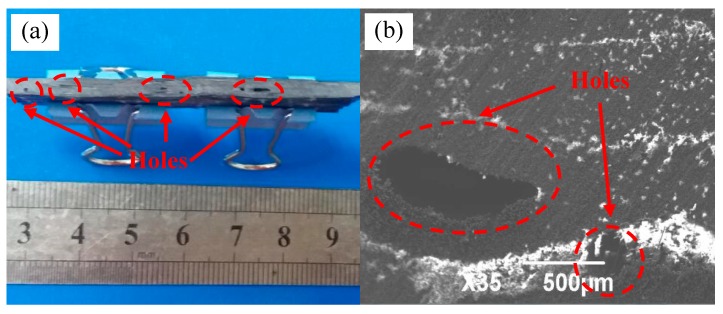
The hole defect of 2D-T700/E44 composite prepared by ICM. (**a**) Macroscopic hole defect; (**b**) Microscopic hole defect.

Figure 7 shows the 2D-T700/E44 composite material with hole defects prepared by this process. Its pressure, temperature and time in pressure heating curing stage are 6.5 MPa, 40 °C and 15 min respectively. As can be seen from the Figure 7a, there are obvious holes in the section of the specimen, whose size and shape are different, and they are uneven distribution. From the Figure 7b, there is a clear bubble-like gap inside the composite, which is closed in shape and forms a cavity inside the sample.

The formation of holes indicates that the composite is not compacted enough, and the porosity is large. The size of porosity is directly related to the bending properties of composites. The higher the porosity is, the worse of the bending strength of materials is [13]. The bending strength of the composite material was tested, and it was 315 MPa, which is not ideal. The process parameters should be controlled and improved to reduce the occurrence of hole defects.

### 3.5. Brittle Fracture of Composite Materials

**Figure 8:**
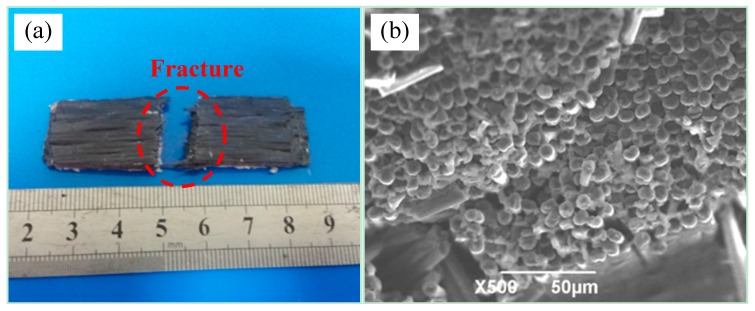
The defect of brittle fracture of 2D-T700/E44 composite prepared by ICM. (**a**) Macrostructure of brittle fracture; (**b**) Micro fracture of brittle fracture.

When the curing temperature is high and the curing time was short, the curing mixed solution is rapidly solidified, and brittle composite material will be formed. When the material is loaded, brittle fracture is likely to occur, resulting in low bending property [15]. Figure 8 shows the fracture morphology of 2D-T700/E44 composite materials with brittle fracture. Its pressure-free heating temperature and time are 130 °C and 80 min, and pressure heating temperature and time are 60 °C and 15 min. As can be seen from the Figure 8a, the fracture is relatively even with only a small amount of fiber pulled out. It can be seen from the microscopic fracture morphology of Figure 8b that the fracture surface of the bent specimen is relatively flat. After the test of bending strength, it was found that the strength of brittle fracture specimen was only 245 MPa, and the property was not satisfactory. In the process test, it is necessary to make the composite solidify uniformly at lower curing temperature and longer curing time, so as to avoid the defects of brittle fracture caused by high temperature.

### 3.6. Composite Materials without Defects 

**Figure 9:**
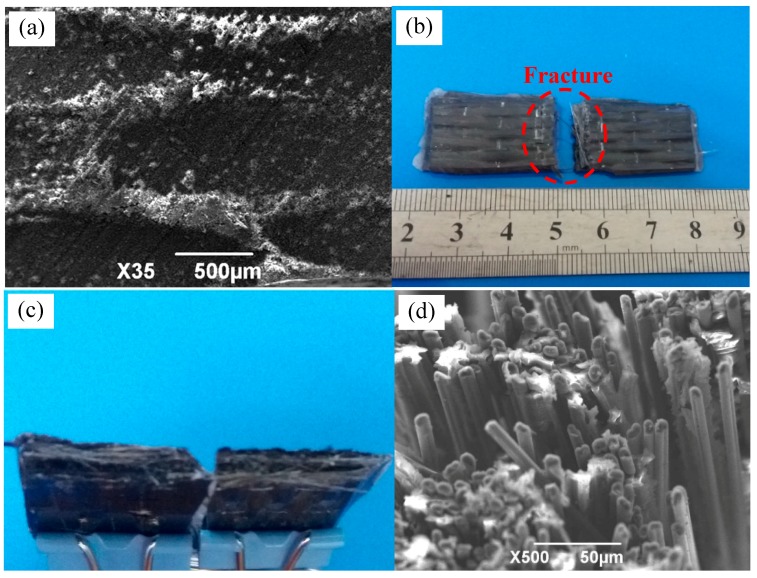
2D-T700/E44 composite without defects prepared by ICM. (**a**) Microstructure impregnation; (**b**) Macrostructure fracture in plane;(**c**) Tridimensional macrostructure fracture; (**d**) Microstructure fracture.

When the process parameters of ICM are appropriate, the defects of 2D-T700/E44 composite can be effectively avoided. The process parameters in Table 1 were used to prepare composite materials and Figure 9a was the impregnation microstructure of the composite materials. There are not obvious defects such as holes and stratification. Figure 9b–d are the bending fracture morphology of the composite material, defects of weak interlaminar binding force, fiber displacement warping and brittle fracture cannot be found. A large number of carbon fiber burrs exists in the section of the specimen damaged by bending load, and the fracture surface is irregular in shape.

From the microscopic fracture in Figure 9d, the resin inside the fracture is evenly distributed and sufficient, and the structure is orderly. There are many holes left by the carbon fibers which are pulled out in the fracture surface. Therefore, 2D-T700/E44 composite material under this condition has stable internal structure and no obvious defects. When the fracture morphology of the composite material is reasonable, carbon fibers and matrix can play roles of reinforcement and transferring load respectively, and the property will be better than that of composites with defects [16,17]. After testing, the bending strength of the composite reaches 630 MPa, which has a significant advantage over the properties of the composite materials with defects.

### 3.7. Property Comparison of Composites under Different Defects

**Figure 10:**
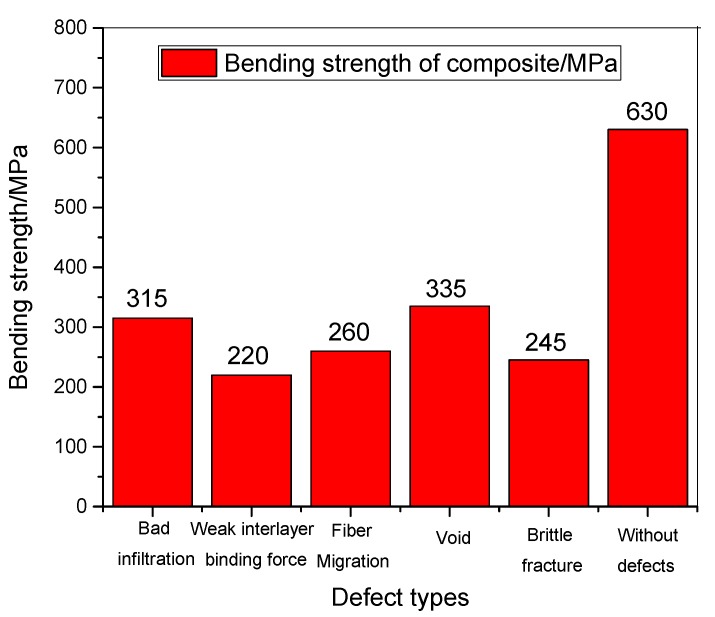
Properties comparison of composites under different defects prepared by ICM.

According to the above analysis, when the parameters of 2D-T700/E44 composite materials prepared by ICM are not reasonable, the defects such as insufficient resin impregnation, weak interlaminar adhesion, fiber displacement and warping, holes and brittle fracture of the composite materials are easy to occur, which will affect the impregnation microstructure, bending properties and fracture morphology of the composite materials. Meanwhile, these defects will affect the preparation of ideal composite materials [18,19]. Bending strength values under different defects in composite materials are presented in Figure 10, and it shows that the bending strength values of the composites are only 220 MPa and 260 MPa and 245 MPa under the defects such as weak interlaminar binding force, brittle fracture of composite material and fiber displacement and warping respectively. They are only 34.9%, 41.3% and 38.9% of the bending strength of 2D-T700/E44 composite without defects. Therefore, the properties are not ideal.

Bending strength values of the composites were 315 MPa and 335 MPa (Figure 10) under the defects such as insufficient resin impregnation and hole. They only reach 50% and 53.2% of the bending strength of 2D-T700/E44 composite without defects, respectively. This is lower than the adverse effect of the previous three defects on the properties, but they are still not ideal. The defects of composite materials need to be reduced and avoided through process improvement and parameter control [20]. Process parameters of curing mixed ratio, curing temperature, pressure and time should be reasonable values [21]. Too large or too small parameter values are unfavorable to the preparation of composite materials with ideal microstructure and properties.

## 4. Conclusions

(1) 2D-T700/E44 composite materials can be prepared by using ICM. When the process parameters of curing mixed ratio, curing temperature, pressure, and time are not controlled properly, the preparation defects such as insufficient impregnation, weak interlaminar binding force, fiber displacement and warping, holes, and brittle fracture of the composite material occur easily.
(2) The effects of different defect forms on the bending properties of CFRP composites are obviously different. Bending strength values of composites under the defects such as weak interlaminar binding force, brittle fracture of composite material and fiber displacement, and warping are only 34.9%, 41.3%, and 38.9%ofthe bending strength of 2D-T700/E44 composite without defects. Bending strength values of the composites under the defects such as insufficient resin impregnation and hole only reach 50% and 53.2% of the bending strength of 2D-T700/E44 composite without defects, respectively.
(3) When the process parameters are reasonable and the defects of composite materials are effectively eliminated, the bending strength can reach 630MPa. If defects occur, the bending property of the composite material will be greatly reduced. Too large or too small parameter values are unfavorable to the preparation of composite materials with ideal microstructure and properties. The defects of composite materials need to be reduced and avoided through process improvement and parameter control.

## Figures and Tables

**Table 1:** Process parameters of improved compression molding (ICM) for preparing 2D-T700/E44 composites.

Curing Mixed Ratio/Mass Ratio	Natural Curing Time/h	Pressure-Free Heating Curing Temperature/°C	Pressure-Free Heating Curing Time/min	Pressure Heating Curing Temperature/°C	Pressure Heating Curing Pressure/MPa	Pressure Heating Curing Time/min
5:1	6	115	110	50	10	25
